# The Association of Obstructive Sleep Apnea and Hypertension

**DOI:** 10.7759/cureus.4858

**Published:** 2019-06-07

**Authors:** Avani R Patel, Amar R Patel, Shivank Singh, Shantanu Singh, Imran Khawaja

**Affiliations:** 1 Internal Medicine, Northern California Kaiser Permanente, Fremont, USA; 2 Internal Medicine, Southern Medical University, Guangzhou, CHN; 3 Pulmonary Medicine, Marshall University School of Medicine, Huntington, USA

**Keywords:** obstructive sleep apnea, hypertension

## Abstract

Obstructive sleep apnea (OSA) is a condition characterized by recurrent episodes of partial or complete upper airway obstruction during sleep. Hypertension (HTN) is defined by the presence of a chronic elevation of systemic arterial pressure above a certain threshold value (≥140 mm Hg systolic or ≥90 mm Hg diastolic). On the surface, OSA and HTN appear very different from one another. Despite this, they share several common risk factors including obesity, male gender, and advancing age. In 2003, the Seventh Joint National Committee (JNC VII) recognized OSA as a secondary cause of HTN. As physicians, our goal is to understand the OSA-HTN association better through academic study regarding its epidemiology, its pathophysiology, and its treatment.

## Introduction and background

Over the years, there has been some overlap between patients of obstructive sleep apnea (OSA) and hypertension (HTN) with about 50% of HTN patients also having concomitant OSA [1]. As physicians, we see more and more patients who have OSA and either develop HTN or have underlying HTN. Because of this, there is a theory that the two conditions may have a causal, bidirectional relationship [1]. This was proven when OSA was named a secondary cause of HTN by the 2003 Joint National Committee (JNC VII) on prevention, detection, evaluation, and treatment of high blood pressure (BP) [2]. A few years later, the 2019 American Heart Association (AHA) reported the conclusion of a meta-analysis of 27 cohort studies [3]. The meta-analysis determined that severe OSA (apnea/hypopnea index ≥30) was associated with increased cardiovascular mortality with a hazard ratio of 2.73 (95% confidence interval [CI], 1.94-3.85) [3]. For this article, the objective is to address the association between OSA and HTN, the epidemiological evidence that supports their causal relationship, the system-specific pathophysiology of OSA that can lead to HTN development, and the treatment of OSA and how it impacts HTN treatment. 

OSA is a condition described as recurrent episodes of upper airway inspiratory collapse during sleep, leading to hypopnea (breathing reduction) or apnea (breathing cessation) episodes that cause transient hypoxemia (low oxygen levels in the blood) and hypercapnia (elevated carbon dioxide levels in the blood) [1]. The patient waking from sleep terminates the apneic and hypopneic episodes [1]. The patient then hyperventilates because of the hypoxemia for a brief period of time [1]. These episodes are key to diagnosing the severity of a patients OSA [1]. The severity is measured by an apnea/hypopnea index (AHI), which measures the number of apnea/hypopnea episodes per hour [1]. The OSA severity is then classified as mild (5-15), moderate (15-30), and severe (30 or more; Table 1) [1]. A patient is diagnosed with OSA when they have an AHI ≥5 events per hour.

**Table 1:** Classification of obstructive sleep apnea Classification of obstructive sleep apnea according to the AHI [1] AHI, apnea/hypopnea index

Classification of Obstructive Sleep Apnea	
Mild	AHI ≥ 5-15 events per hour
Moderate	AHI ≥ 15-30 events per hour
Severe	AHI ≥ 30 or more events per hour

HTN is a condition that refers to a sustained increase in BP beyond certain systolic (SBP) and diastolic blood pressure (DBP) levels. The majority of current definitions define HTN as SBP ≥140 mm Hg and/or DBP ≥90 mm Hg [4]. HTN is categorized into essential and secondary. According to an earlier research study by Carretero and Oparil, essential HTN has been defined as high BP in which secondary causes such as renal disease, pheochromocytoma, aldosteronism, or other causes of secondary HTN are not present [5]. The current guidelines for HTN diagnosis are listed in Table 2. The divisions are normal, prehypertension, stage one HTN, and stage two HTN [2]. 

**Table 2:** The 2003 Joint National Committee Seventh Report on Blood Pressure Classification Current blood pressure classification according to the 2003 Joint National Committee (JNC VII) on prevention, detection, evaluation, and treatment of high blood pressure [2]

2003 Joint National Committee Seventh Report on Classification of Blood Pressure in Adults			
Classification	Systolic Blood Pressure		Diastolic Blood Pressure
Normal	<120 mm Hg	and	<80 mm Hg
Prehypertension	120-139 mm Hg	or	80-89 mm Hg
Stage 1 Hypertension	140-159 mm Hg	or	90-99 mm Hg
Stage 2 Hypertension	≥60 mm Hg	or	≥100 mm Hg

## Review

Epidemiology of OSA and HTN

OSA is a highly prevalent sleep disorder that is estimated to affect 15% to 24% of all adults, but that the number is believed to be incorrect because OSA is still greatly underdiagnosed [6]. In 2017, a research study tried to determine the prevalence of OSA in the general adult population and how it varied between different sub-groups [7]. They examined 24 studies and divided the results according to age and AHI. With an AHI ≥5 events per hour, the overall prevalence was between 9% and 38% in the general adult population [7-9]. For men having OSA with an AHI ≥5, the prevalence ranged from 13% to 33% [9-10]. For women having OSA with an AHI ≥5, the prevalence ranged from 6% to 19% [9-10]. It was also determined that in some advanced age groups, the OSA prevalence was about 84% [11]. It was even higher in the men, with a 90% prevalence [11]. Moderate OSA (AHI ≥15) had an adult population prevalence that ranged from 6% to 17% but was 36% in the above 60 years age group [3,8-9,11-12]. Another report stated that a meta-analysis of 27 cohort studies determined that the mild OSA hazard ratio was 1.19 (95% CI, 0.86-1.65), the moderate OSA hazard ratio was 1.28 (95% CI, 0.96-1.69), and the severe OSA hazard ratio was 2.13 (95% CI, 1.68-2.68) and was associated with an all-cause mortality in a dose-response fashion [3]. 

According to a report from AHA, between 2011 and 2014, the prevalence of HTN in the United States adults was 45.6% (95% CI, 43.6% to 47.6%) [3]. This was calculated using the new BP classification from the 2017 American College of Cardiology/AHA guidelines (Table 3) [3,13]. This was in comparison to JNC VII [3]. Based on the 2003 JNC VII classification, the prevalence of HTN was 31.9% (95% CI, 30.1% to 33.7%) in the United States adults [2]. 

**Table 3:** The 2017 American College of Cardiology Classification of High Blood Pressure Before diagnosing a patient with hypertension, physicians must base the diagnosis on the average value of more than two BP readings obtained on more than two different occasions [13]. BP, blood pressure

2017 American College of Cardiology/AHA Classification of High Blood Pressure		
Classification	Systolic Blood Pressure	Diastolic Blood Pressure
Normal	< 120 mm Hg	and < 80 mm Hg
Elevated Blood Pressure	120-129 mm Hg	and < 80 mm Hg
Hypertension Stage 1	130-139 mm Hg	or 80-89 mm Hg
Hypertension Stage 2	≥ 140 mm Hg	or ≥ 90 mm Hg

**Figure 1:**
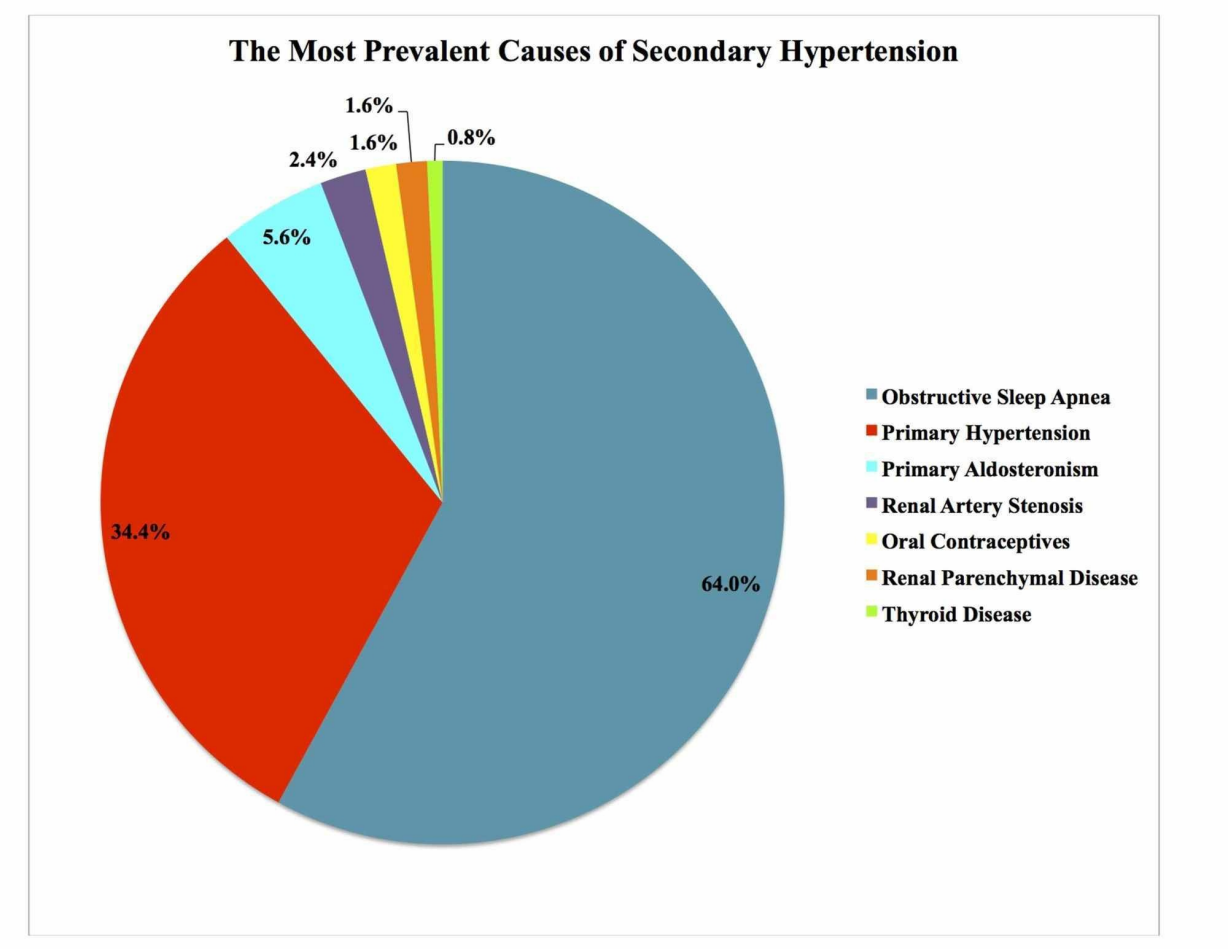
The prevalence of secondary causes of hypertension A created pie chart representing a 1985 study examining the prevalence of secondary causes of hypertension associated with resistant hypertension in a cohort of 125 patients from Brazil. From this study, it was determined that OSA was the most prevalent secondary cause of resistant hypertension [18].

Past research studies have been successful in demonstrating epidemiological evidence of the OSA-HTN relationship. They have determined that OSA is not only a predisposing factor for HTN but there is also a greater incidence of OSA in hypertensive patients [14-15]. OSA is estimated to have a prevalence of 30% to 50% in HTN patients [16-17]. In comparison, the prevalence of HTN in OSA patients is between 30% and 70% [17]. This is because OSA is under-diagnosed [16-17]. In a previous paragraph, it was mentioned that the JNC VII stated that OSA was a secondary cause of HTN. This was further proven by a 2011 cohort study done in Brazil with 125 patients that determined that OSA was the most prevalent secondary cause of elevated BP in patients [18]. The figure below represents the most prevalent causes of secondary HTN (Figure 1).

Pathophysiology

**Figure 2:**
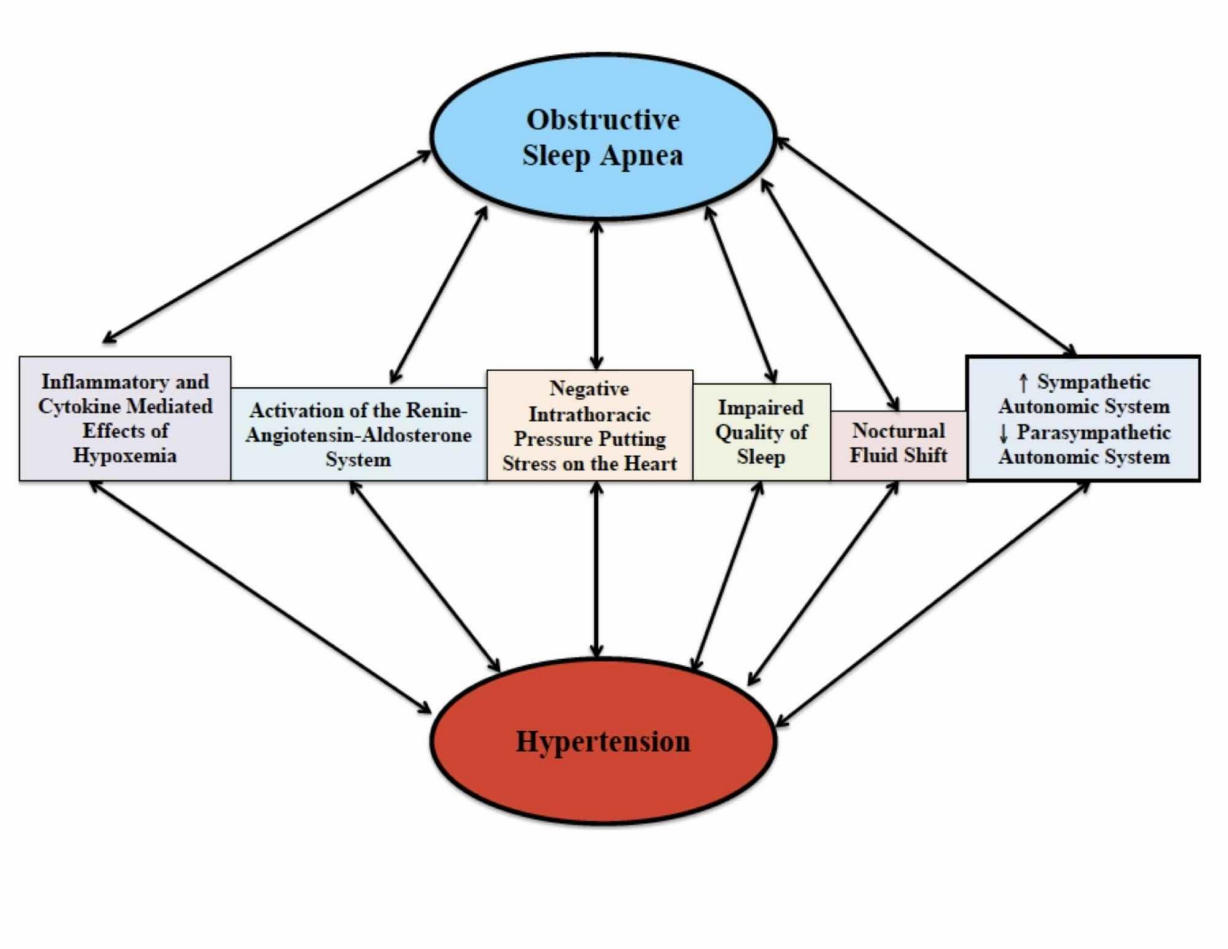
The pathophysiological mechanisms shared between obstructive sleep apnea and hypertension Flow chart representing the shared pathophysiological mechanisms between OSA and HTN OSA, obstructive sleep apnea; HTN, hypertension

OSA and HTN are both multifactorial diseases [19]. They share many of the same risk factors (obesity, male gender, and advancing age) [6]. Because of this, and the fact that OSA is the most prevalent secondary cause of HTN, both also share many pathophysiological mechanisms that link them together [18-19]. By understanding these mechanisms as determined by previous research, the development of HTN in OSA cases, and the overall increased risk of cardiovascular disease can be better understood. The figure given below has been created representing different pathophysiological mechanisms linking the two conditions (Figure 2). 

Sleep Inefficiency Due to OSA

Impaired sleep quality (sleep inefficiency and shorter sleep duration) can lead to OSA development [19]. There is decreased sympathetic and increased parasympathetic activity during non-rapid eye movement (NREM) sleep [1]. NREM sleep consists of the majority of sleep time and contributes to the normal circadian variation of BP that leads to a “dipping” of both systolic and diastolic BP at night (decreases by 10% to 15%) [20-21]. NREM sleep is followed by rapid eye movement (REM) sleep, which has increased sympathetic activity that leads to transient BP surges [1]. REM sleep is also characterized by generalized skeletal muscle atonia causing an airway collapse in OSA patients especially [1]. This leads to the exacerbation of potential apneic episodes and intensifies the patient’s nocturnal sympathetic hyperactivity [1]. A 2012 longitudinal study found that chronic insomnia (*p* = 0.004) and short sleep duration (*p* = 0.003) were both significantly associated with HTN, whereas poor sleep was not (*p* = 0.756) [22].

Nocturnal Fluid Shift in OSA

Another significant pathophysiological mechanism is nocturnal fluid redistribution [19]. During the night, the fluid accumulated in the legs will redistribute to the neck. This is especially significant for OSA and HTN patients in that the reduction of the mean upper airway cross-sectional area can intensify hypopnea/apnea episodes and resultant hypoxia, which will ultimately lead to transient BP surges [19]. Friedman et al. (2013) set forth to examine this theory [23]. The theory was proven significant and it was also determined that the upper airway reduction was seen more in resistant HTN patients as compared to controlled HTN patients [23]. OSA also has a prevalence of 83% in resistant HTN patients [24]. Resistant HTN is defined as a BP ≥140/90 mmHg, while the patient is taking three or more antihypertensive drugs, all of them titrated to the maximum recommended dose [24]. HTN patients would be prone to an increased nocturnal fluid redistribution which worsens their OSA by leading to transient BP surges, which later causes resistant HTN [23].

The Autonomic System Counterregulatory Mechanisms Against Apneic Episodes

OSA patients have apneic episodes caused by airway collapse during sleep [1]. These episodes lead to transient hypoxemia and hypercapnia that activate the sympathetic autonomic system and down-regulate the parasympathetic autonomic system [1,25]. The increased activation of the sympathetic system leads to an increase in catecholamine levels, causing a rise in heart rate and BP that persists into the next day [26]. The rise is most prominent during post-apneic hyperventilation going as high as 240/130 mm Hg [27-28]. Over time, this sympathetic stimulation can lead to the development of HTN in an OSA patient. 

The Inflammatory and Cytokine-mediated Effects of Hypoxemia

OSA causes intermittent nocturnal hypoxemia and hypercapnia that causes oxidative stress and inflammation [19]. The oxidative stress acts like an ischemic reperfusion injury, leading to the release of reactive oxygen species, inflammatory cytokines (hs-CRP, IL-1, IL-8, IL-6, TNF-α, Rantes, and sICAM), and vasoactive substances [29-30]. This leads to an increase in endothelin, a decrease in nitric oxide, vasoconstriction, and endothelial dysfunction [1, 29-30]. Overall, oxidative stress can lead to increased cardiovascular risk. 

Negative Intrathoracic Pressure Putting Stress on the Heart

OSA causes intermittent negative intrathoracic pressure in patients [1]. This pathophysiology combined with OSA nocturnal catecholamine surges can put profound mechanical stress on the heart which can lead to left ventricular hypertrophy and atrial remodeling, thus increasing the risk of heart failure and arrhythmia formation [31]. 

The Renin-Angiotensin-Aldosterone System

**Figure 3:**
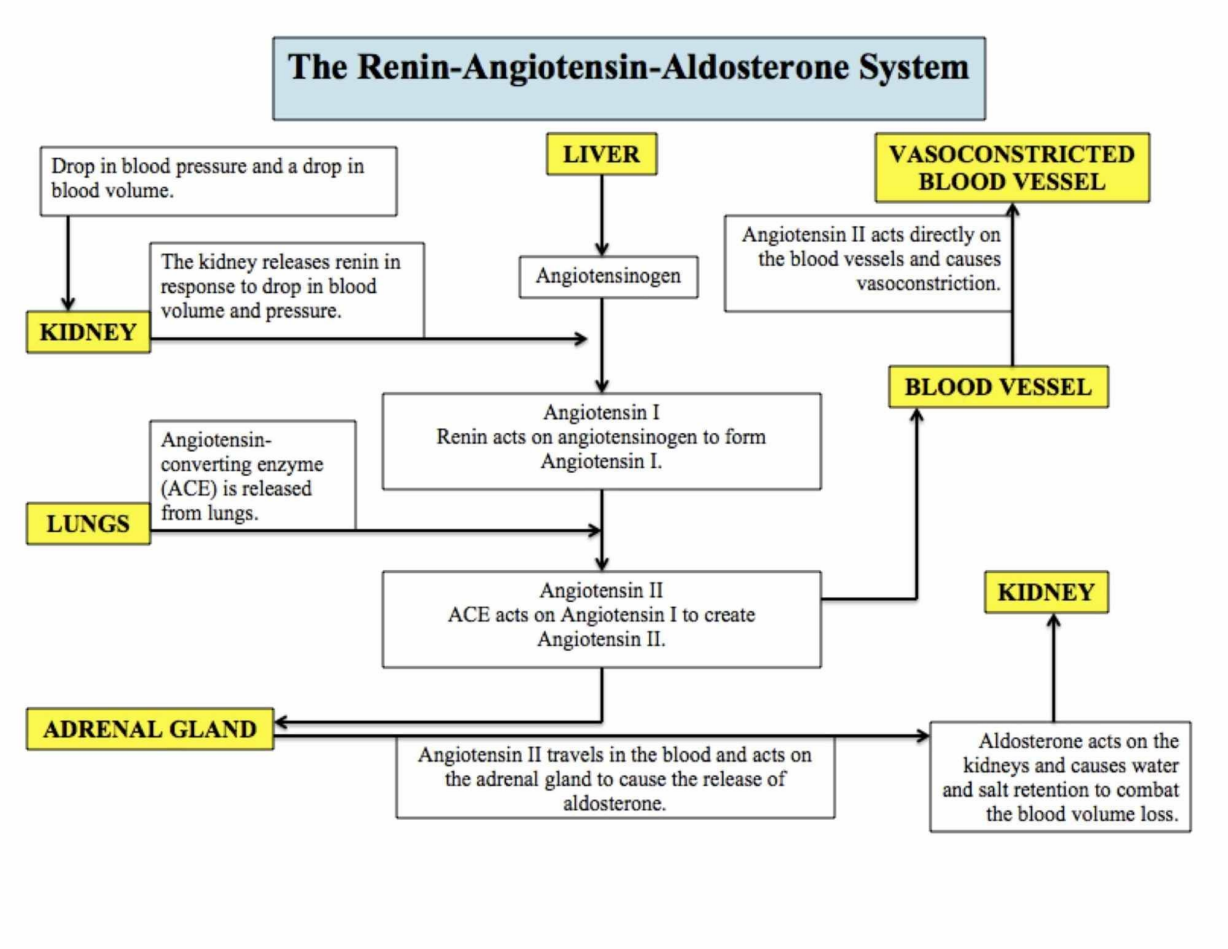
The renin-angiotensin-aldosterone system Flow chart representing the renin-angiotensin-aldosterone system and the organs and hormones involved in its regulation

OSA causes hypoxemia leading to an activation of the renin-angiotensin-aldosterone system (RAAS) [1]. RAAS stimulation increases renin and aldosterone levels (Figure 3) [32]. A 2016 meta-analysis of 13 studies determined that OSA patients had elevated angiotensin II levels compared to control subjects and OSA patients with HTN had higher aldosterone levels compared to normotensive OSA patients [33]. Continuous positive airway pressure (CPAP) therapy is the gold standard of treatment for OSA [1]. CPAP therapy is associated with a down-regulation of RAAS activity, leading to consequent BP reduction [34]. From the reduction of BP and the markers, it was concluded that RAAS had a causal role in OSA-mediated HTN [33]. Increased aldosterone caused by RAAS activation can also contribute to fluid retention seen in HTN, which leads to more rostral fluid displacement and an increase in upper airway obstruction [1,35]. This will, in turn, worsen the patient’s hypoxemia and the pathophysiological cycle will continue. 

Management

With OSA and HTN, the goals of initial evaluation are to determine the patient’s baseline, evaluate for target organ damage, screen for potentially curable causes, identify risk factors that are present, determine the prognosis, and choose a therapy that is specific to the patient’s needs [5]. A complete history and physical examination should be done [5]. The patient should also undergo extensive laboratory investigations such as a urine analysis, complete blood count, blood chemistry (potassium, sodium, creatinine, fasting glucose, total and high-density lipoprotein or HDL cholesterol), a 12-lead ECG, creatinine clearance, 24-hour urinary protein, serum uric acid levels, serum calcium, glycosylated hemoglobin, fasting lipid panel, an echocardiography, and plasma renin activity/aldosterone measurements [5]. 

CPAP Therapy

For all cases of OSA, CPAP remains the main therapy utilized but its effect on BP reduction has not been proven to be completely effective [1]. In previous clinical trials, CPAP therapy helped reduce the nocturnal sympathetic catecholamine release and their resulting BP surge, but overall BP reduction was not very significant (1.3-3 mm Hg) [36-38]. Despite the modest BP reduction seen in clinical studies, CPAP therapy improves cardiovascular and cerebrovascular health in patients by reducing stroke mortality by 6% to 8% and ischemic heart disease mortality by 4% to 5% [2,39].

Lifestyle Modifications

Obesity is one of the few risk factors commonly seen in both OSA and HTN. Because of this, a reduction in weight can help reduce the OSA severity and BP in an HTN patient. A 2000 cohort study found that a weight gain of 10% led to a 32% increase in AHI and a six-fold increase in the odds of developing moderate to severe OSA [40]. In addition, the Wisconsin study involved was also able to determine that a 10% weight loss would lead to a 26% AHI decrease [40]. Because OSA is the most prevalent secondary cause of HTN, any decrease in its severity will directly affect HTN severity or development [18].

Antihypertensive Drugs

Antihypertensive medications can be prescribed to all patients with HTN who have mild to moderate OSA (who do not require CPAP therapy) [1]. They can also be given to patients of severe OSA who are either non-tolerant or non-compliant with CPAP therapy [1]. HTN in OSA can occur from catecholamine release from the activated sympathetic system or from the RAAS system activation [1]. Because of this, beta-blockers and aldosterone antagonists may be the best treatment options as they act on these mechanisms [1]. The aldosterone antagonist spironolactone is considered very effective for decreasing the severity of OSA [41]. Another antihypertensive medication called atorvastatin is known to reduce inflammation, which helps reduce the patient’s cardiovascular risk [42].

Oral Appliances

In mild to moderate OSA, oral appliances can be recommended as an alternative treatment to CPAP [1]. A meta-analysis of seven studies (399 OSA patients involved) found that treatment with oral appliances was more beneficial for BP reduction than CPAP therapy [43]. The average drop in the systolic BP and diastolic BP was 2.7 mm Hg (95% CI, 0.8-4.6; *P *= 0.04) and 2.7 mm Hg (95% CI, 0.9-4.6; 𝑃 = 0.004 ), respectively [43].

Upper Airway Surgery

Upper airway surgery is also a treatment option for OSA patients who require BP reduction [1]. These options include tonsillectomy and uvulopalatopharyngoplasty (UPPP) [1]. A 2010 randomized controlled trial determined that modified UPPP significantly improved sleepiness, nocturnal respirations, and quality of life [44]. The trial also determined that the BP was reduced significantly after surgery in a select group of patients with moderate to severe OSA [44].

## Conclusions

More and more OSA patients present or need treatment for co-existing HTN. The OSA airway collapse leads to the BP being driven up, and without treatment, the patient continues in a self-perpetuating pathophysiological cycle that leads to an increase in their cardiovascular and cerebrovascular risk. Although enormous collected data and treatments are available for OSA requiring BP reduction, more strategies are critically needed. The only way to make this happen is to create a path for more research and larger clinical trials. As physicians, we must spearhead this cause because more and more of our patients will come in with this condition. 
